# Breakthrough *Pediococcus acidilactici* and *Leuconostoc lactis* bacteraemia with elevated meropenem minimum inhibitory concentrations during broad-spectrum antimicrobial therapy in a patient with advanced pancreatic cancer: A case report and review of the literature

**DOI:** 10.1016/j.idcr.2026.e02692

**Published:** 2026-07-18

**Authors:** Aung Du, Sudha Pottumarthy-Boddu, Dipna Martin-Robins, Jacqueline Mazzer, Blathine Tierney, Shannyn George, Andrew Hart, Eliza Briggs, Andrew Dean, Arindam Chakravorty

**Affiliations:** a Sir Charles Gairdner Hospital, Nedlands, Western Australia, Australia; b St John of God Hospital, Subiaco, Western Australia, Australia; c Australian Clinical Labs, Osborne Park, Western Australia, Australia; d Rockingham General Hospital, Cooloongup, Western Australia, Australia; e Kalamunda Hospital, Kalamunda, Western Australia, Australia; f WA Medical Oncology, Subiaco, Western Australia, Australia; g Fiona Stanley Hospital, Murdoch, Western Australia, Australia; h The University of Western Australia, Crawley, Western Australia, Australia

**Keywords:** Leuconostoc, Pediococcus, Bacteraemia, Immunosuppression, Carbapenem, Resistance

## Abstract

*Pediococcus acidilactici* and *Leuconostoc lactis* are bacteria commonly found in dairy and probiotic products that are intrinsically resistant to glycopeptides. Although historically regarded as organisms of low pathogenicity, there are increasing case reports with invasive infections, primarily in immunocompromised hosts with compromised bowel mucosa. However, concurrent bacteraemia with elevated meropenem minimum inhibitory concentrations (MICs) in both organisms has not been previously described. Here we present the first case, occurring in a patient with advanced pancreatic cancer, peritoneal and hepatic metastases, on a background of a polymicrobial liver abscess. Breakthrough bacteraemia with *P. acidilactici* and *L. lactis* developed despite treatment with meropenem and teicoplanin. Both organisms appeared as streptococci/enterococci on Gram stain, with definitive identification of culture isolates using matrix-assisted laser desorption/ionisation time-of-flight mass spectrometry (MALDI-TOF-MS). Retrospective meropenem susceptibility testing demonstrated MICs of > 32 mg/L for *P. acidilactici* and 16 mg/L for *L. lactis*. The case highlights two key messages. First, although *P. acidilactici* and *L. lactis* mimic streptococci/enterococci on Gram stain, empirical glycopeptide therapy – usually reliable for these organisms – would be ineffective, and elevated meropenem MICs suggest carbapenems may also be unreliable. Therefore, early species-level identification by MALDI-TOF-MS and prompt susceptibility testing are essential. Second, prolonged and/or broad-spectrum antimicrobial exposure selects for pathogens with reduced susceptibility, particularly in immunocompromised hosts with disrupted gut mucosa and unachievable source control. As patients with advanced malignancy receive prolonged courses of broad-spectrum antimicrobials, infections with *Pediococcus* spp. and *Leuconostoc* spp. may be encountered more frequently and deserve consideration in this setting.

## Introduction

*Pediococcus acidilactici* and *Leuconostoc lactis* are Gram-positive, catalase-negative cocci found in dairy and probiotic products that can transiently colonise the human gastrointestinal tract [1], [2], [3], [4]. Although traditionally regarded as low-pathogenicity organisms, they are increasingly recognised as opportunistic pathogens capable of causing invasive infections [3], [5]. Both appear as Gram-positive cocci in chains, resembling streptococci or enterococci on initial Gram stain and display intrinsic resistance to glycopeptides [6], with reliable species-level identification requiring matrix-assisted laser desorption/ionisation time-of-flight mass spectrometry (MALDI-TOF-MS) [7]. Before definitive confirmation, treating clinicians are guided by the preliminary Gram stain, which typically prompts glycopeptide therapy. However, glycopeptide resistance in these organisms may remain undetected at this stage, resulting in inadequate empirical coverage. Furthermore, emerging evidence – including the present case – suggests that carbapenems may also be unreliable, as elevated minimum inhibitory concentrations (MICs) have been documented.

Invasive infections with *P. acidilactici* and *L. lactis* are largely confined to immunocompromised hosts, and several recurring risk factors have been identified. Disruption of the gastrointestinal mucosal barrier, whether due to surgery or malignancy, is thought to provide a portal of entry, enabling bacterial translocation from the gastrointestinal lumen into the bloodstream [2], [3], [8]. Additional risk factors include malignancy, immunosuppression (diabetes, immunosuppressive drugs, and chemotherapy), and the presence of central venous catheters [2], [3], [5], [9], [10], [11]. Prior antimicrobial exposure is another commonly reported feature, reinforcing the role of selection pressure in the emergence of these organisms in vulnerable hosts [5], [10], [12].

To date, no cases of concurrent bacteraemia with *P. acidilactici* and *L. lactis* with elevated meropenem MICs have been reported. Here, we present the first such case in a man with advanced pancreatic cancer who, after a three-month admission for recurrent bacteraemia and a polymicrobial liver abscess, developed breakthrough bacteraemia with both organisms despite ongoing treatment with meropenem and teicoplanin.

## Case report

A 39-year-old man with advanced pancreatic adenocarcinoma with liver and peritoneal metastases was admitted to a private hospital in Western Australia following a febrile episode at home. His medical history includes previous recurrent gastrointestinal bleeds, prior hepatic abscess, hypertension, well-controlled asthma and a previous pancreaticoduodenectomy in January 2024. Initial blood cultures grew *Escherichia coli (E. coli)*; initial empirical piperacillin-tazobactam was de-escalated to amoxycillin-clavulanate on day 4 following return of antimicrobial susceptibility results and ceased on day 15. A chronological summary of events is provided in Table 1.

**Table 1:** Timeline of events. Chronology of clinical and microbiological events and antimicrobial management. Day 1 corresponds to the date of hospital admission.

Day	Clinical event	Treatment event
1	Febrile, blood cultures obtained	Commenced IV piperacillin-tazobactam
2	Preliminary Gram stain reported Gram-negative bacilli	
3	Blood cultures grew *Escherichia coli*	
4		Replaced IV piperacillin-tazobactam with IV amoxycillin-clavulanate
11		Stepped down from IV to oral amoxycillin-clavulanate
12	Chemotherapy with folinic acid, fluorouracil, irinotecan and oxaliplatin (FOLFIRINOX) regimen	
15		Ceased oral amoxycillin-clavulanate
16	Febrile and tachycardic, blood cultures obtained	Recommenced IV amoxycillin-clavulanate
17	Preliminary Gram stain reported Gram-negative bacilli	Replaced IV amoxycillin-clavulanate with IV meropenem
18	Blood cultures grew ESBL producing *Escherichia coli*	
20	Infusaport removed due to presumed source of infection, peripherally inserted central catheter placed	
25	Infusaport site swab grew *Staphylococcus capitis*	Remained on IV meropenem
26	Chemotherapy with folinic acid, ralitrexed, irinotecan and oxaliplatin regimen	
31		Ceased IV meropenem
41	Chemotherapy with nab-paclitaxel and gemcitabine	
44	Tachycardic, blood cultures obtained. Imaging evidence of liver lesions (metastasis versus abscess)	
45	Preliminary Gram stain reported Gram-negative bacilli	Recommenced IV meropenem
46	Blood cultures grew *Escherichia coli, K. pneumoniae* and ESBL-producing *Escherichia coli*	
61	Febrile and tachycardic, blood cultures obtained	
62	Preliminary Gram stain reported Gram-positive cocci resembling streptococci/enterococci. Imaging evidence of a liver abscess	Commenced IV vancomycin, IV meropenem ongoing
63	Blood cultures grew *Enterococcus raffinosus and Enterococcus faecium*. Recurrent febrile episodes and concerns of glycopeptide resistance prompted change from vancomycin to daptomycin	Replaced IV vancomycin with IV daptomycin. IV meropenem ongoing.
66	Blood cultures repeated. Poor venous access necessitated subcutaneous route.	Replaced IV daptomycin with SC teicoplanin, IV meropenem ongoing
67	Preliminary Gram stain reported Gram-positive cocci resembling streptococci/enterococci	IV anidulafungin commenced, SC teicoplanin and IV meropenem ongoing
68	Blood cultures grew *Enterococcus faecium, Enterococcus raffinosus* and *Nakaseomyces glabratus*. Liver abscess confirmed and drained.	
73	Liver abscess aspirate grew *Enterococcus faecium*, E*nterococcus raffinosus* and *Nakaseomyces glabratus*	
88	Febrile and tachycardic, blood cultures obtained.	
89	Preliminary Gram stain reported Gram-positive cocci resembling streptococci/enterococci	
90	Blood cultures grew *Leuconostoc lactis*	
91	Blood cultures grew *Pediococcus acidilactici*	
93	Penicillin, ampicillin and vancomycin susceptibility results for *Leuconostoc lactis* and *Pediococcus acidilactici* reported	
96	Shared decision-making to prioritise comfort-focused care	Ceased subcutaneous teicoplanin, IV anidulafungin and IV meropenem
103	Death	

Abbreviations: Intravenous, IV; subcutaneous, SC; extended-spectrum beta-lactamase, ESBL.

Over the next six weeks, his course was complicated by recurrent bacteraemia with several Enterobacterales. On day 18, extended-spectrum beta-lactamase (ESBL) *E. coli* was isolated in blood cultures and his subclavian Infusaport (inserted 2024) was removed as a potential intravascular source of infection. He completed a course of meropenem from day 17 until 31. Bacteraemia recurred on day 46 with ESBL *E. coli*, non-ESBL producing *E. coli* and *Klebsiella pneumoniae,* prompting re-initiation of meropenem.

On day 61, he became febrile and tachycardic despite meropenem therapy, and blood cultures were taken. A liver abscess was identified the following day, though aspiration was deferred due to thrombocytopenia. Preliminary microscopy demonstrating Gram-positive cocci from positive blood culture bottles prompted commencement of vancomycin on day 62 and polymicrobial bacteraemia with *Enterococcus raffinosus* and *Enterococcus faecium* was confirmed on day 63. Given recurrent fevers and concern for glycopeptide resistance, vancomycin was switched to daptomycin on day 63 and, due to limited venous access, to subcutaneous teicoplanin on day 67. Following supportive transfusions and recovery of platelet count, the liver abscess was aspirated on day 68; demonstrating polymicrobial growth of *Enterococcus raffinosus*, *Enterococcus faecium* and *Nakaseomyces glabratus* (formerly *Candida glabrata*). Anidulafungin was commenced, in addition to meropenem and teicoplanin. All enterococcal isolates were susceptible to vancomycin and teicoplanin.

On day 88, the patient reported palpitations and mild dyspnoea. He was febrile (38.9°C), tachycardic (148 bpm) and hypertensive (160/90 mmHg). His respiratory rate was 16 breaths per minute, with an oxygen saturation of 100% on room air. Cardiorespiratory examination was unremarkable. Moderate ascites was present at baseline and abdomen was soft on palpation. Investigations showed haemoglobin 101 g/L, leucocytosis (19.5 ×10^9^/L), neutrophilia (17.2 ×10^9^/L), thrombocytopenia (76 ×10^9^/L), elevated C-reactive protein (153 mg/L) and creatinine of 68 micromol/L. One set of blood cultures comprising of one aerobic and one anaerobic bottle, was obtained. Despite ongoing broad-spectrum therapy, the aerobic bottle flagged positive on day 89 for Gram-positive cocci resembling streptococci or enterococci, with definitive identification of *Leuconostoc lactis* and *Pediococcus acidilactici* reported on day 90 and 91, respectively. The anaerobic bottle remained negative throughout the incubation period. The patient did not receive parenteral nutrition or probiotic supplements during this admission. Susceptibility testing for ampicillin/amoxycillin, penicillin and vancomycin was reported on day 93, with MICs shown in Table 2 interpreted in accordance with Clinical and Laboratory Standards Institute (CLSI) breakpoints [13]. Antimicrobial therapy continued until day 96, when care was transitioned to comfort measures per the patient’s wishes. He died on day 103. Retrospective susceptibility testing of *P. acidilactici* and *L. lactis* isolates against meropenem was performed on day 132 using Etest (bioMérieux) [14], yielding MICs of > 32 mg/L and 16 mg/L, respectively.

**Table 2:** Antimicrobial susceptibility results for *Pediococcus acidilactici* and *Leuconostoc lactis*. Susceptibility breakpoints were interpreted in accordance with Clinical and Laboratory Standards Institute (CLSI) breakpoints [13].

	*Pediococcus acidilactici*		*Leuconostoc lactis*	
	Reported MIC	CLSI breakpoint for *Pediococcus* spp.	Reported MIC	CLSI breakpoint for *Leuconostoc* spp.
Penicillin	1.0 mg/L	≤ 8 mg/L	0.125 mg/L	≤ 8 mg/L
Ampicillin/amoxycillin	4.0 mg/L	≤ 8 mg/L	0.25 mg/L	≤ 8 mg/L
Vancomycin	Resistant	Not applicable	Resistant	Not applicable

Abbreviations: miligrams per litre, mg/L; sensitive, S; minimum inhibitory concentration, MIC.

## Discussion

*P. acidilactici* and *L. lactis* bacteraemia is an emerging clinical challenge, with most reported cases occurring in patients with underlying malignancy or a history of abdominal surgery [2], [15]. A search of PubMed, EMBASE, Scopus and the Cumulative Index to Nursing and Allied Health Literature (CINAHL) was conducted to identify, to the best of our knowledge, all previously published cases of *P. acidilactici* and *L. lactis* bacteraemia up to 15 May 2026. Non-English publications, reports lacking species-level identification and case series with insufficient clinical detail were excluded. The identified cases are summarised in Appendix A *(P. acidilactici)* and Appendix B *(L. lactis).*

Recurring risk factors across the identified cases mirrored those in our patient. Among the 16 identified cases of *P. acidilactici* bacteraemia, an underlying malignancy was present in 5 (31%), abdominal surgery or gastrointestinal pathology with compromised mucosal integrity in 10 (63%), an indwelling central venous catheter in 4 (25%) and additional immune-compromising comorbidities (diabetes mellitus, immunosuppressant drugs and/or chemotherapy) in 5 (31%). A broadly similar risk profile was observed among 18 cases of *L. lactis* bacteraemia, with malignancy in 6 (33%), abdominal surgery or gastrointestinal pathology in 7 (39%), a central venous catheter in 8 (50%) and similar immune-compromising comorbidities in 7 (39%). Collectively, these findings reinforce that *P. acidilactici* and *L. lactis* bacteraemia predominantly affect hosts with malignancy, immunosuppression, disrupted mucosal barriers or indwelling vascular devices [2], [15]. The Gram stain morphology of these organisms represents an important diagnostic consideration. Both *P. acidilactici* and *L. lactis* present as Gram-positive cocci in chains, resembling streptococci or enterococci [16], [17], an appearance that commonly prompts empirical glycopeptide therapy despite their intrinsic resistance, conferred by a terminal D-alanyl-D-lactate peptidoglycan moiety [6]. In our case, the preliminary Gram stain offered no indication that ongoing teicoplanin was ineffective, and definitive identification required an additional 24 h, leaving an immunocompromised host with bacteraemia without effective antimicrobial cover. This illustrates the value of expedited species-level identification, in this case by MALDI-TOF-MS, which reliably distinguishes these organisms from their Gram-positive mimics upon culture [7]. Where MALDI-TOF-MS is unavailable, evidence of ongoing bacteraemia despite glycopeptide therapy in an immunocompromised patient should prompt consideration of *Pediococcus* spp. or *Leuconostoc* spp. infection, with appropriate empirical cover pending definitive identification.

Empirical escalation to carbapenems, while clinically common, may not always provide adequate cover. Carbapenem activity against *P. acidilactici* and *L. lactis* has been inconsistently described, with CLSI breakpoints only available for *Pediococcus* species against imipenem [13]. In an analysis of 90 *P. acidilactici* strains, Maugein et al. reported imipenem MICs ranging from 0.12 to 1 mg/L [18] and a case report by Iwen et al. [9] documented a meropenem MIC of 2 mg/L. Carbapenem resistance in *Leuconostoc* species is better recognised [13]. In an analysis of 20 blood isolates, Lee et al. [15] reported meropenem MICs of 1–16 mg/L and doripenem MICs of 0.5–16 mg/L. Case reports of *L. lactis* bacteraemia have demonstrated similar findings, with Yang et al. [10] reporting a meropenem MIC of 4 mg/L and an ertapenem MIC of ≥ 8 mg/L, and Matsuda et al. [19] reporting a doripenem MIC of 6 mg/L. Our findings – meropenem MICs of > 32 mg/L for *P. acidilactici* and 16 mg/L for *L. lactis* – substantially exceed previously published MICs for the former and represent the upper range of those reported for the latter – suggest that carbapenems cannot be assumed to be effective against *P. acidilactici* and *L. lactis* bacteraemia. As both isolates retained penicillin susceptibility, we postulate that the elevated meropenem MICs reflect efflux pumps or altered penicillin-binding proteins rather than beta-lactamase production, a mechanism not previously characterised in these organisms. Reported antimicrobial regimens used to achieve successful treatment have varied considerably (Appendices A and B).

The emergence of unusual resistant organisms is likely related to the selection pressure exerted by sustained and/or broad-spectrum antimicrobial therapy, with such exposure documented in 30 of 35 (86%) reported cases in the three months preceding bacteraemia. Heinz et al. [20] attributed the selection of *P. acidilactici* to prolonged broad-spectrum antimicrobial use, while Yang et al. [10] proposed that long-term vancomycin administration facilitated the overgrowth of *L. lactis* through selective suppression of other vancomycin-susceptible Gram-positive organisms. Our patient illustrates this phenomenon clearly. By the time *L. lactis* and *P. acidilactici* were isolated on days 90 and 91, respectively, he had received three months of near-continuous antimicrobials, including piperacillin-tazobactam, amoxycillin-clavulanate, vancomycin, daptomycin, teicoplanin, anidulafungin and extended courses of meropenem.

This case highlights two practical implications for infectious disease practice. First, *Pediococcus* spp. and *Leuconostoc* spp. should be suspected in high-risk immunosuppressed patients when Gram-positive cocci are reported on preliminary Gram stain and the patient is on adequate antimicrobial cover; early MALDI-TOF-MS identification and susceptibility testing are essential, as glycopeptides are inactive and carbapenems cannot be assumed reliably effective. Second, prolonged or broad-spectrum antimicrobial use may select for these organisms; therefore, a history of such exposure should heighten clinical suspicion for *Pediococcus* spp. or *Leuconostoc* spp. bacteraemia in the appropriate host. In our patient, the combination of malignancy, chemotherapy, increased gastrointestinal permeability, the presence of a central venous catheter and prolonged antimicrobial therapy collectively led to *P. acidilactici* and *L. lactis* bacteraemia.

## Conclusion

We report the first case of concurrent *P. acidilactici* and *L. lactis* bacteraemia with elevated meropenem MICs in a patient with advanced pancreatic adenocarcinoma, prolonged broad-spectrum antimicrobial therapy, unattainable source control and presumed bacterial translocation from the gastrointestinal tract. Two key messages emerge for similar high-risk patients. First, *Pediococcus* spp. and *Leuconostoc* spp. mimic streptococci/enterococci on Gram stain, risking ineffective glycopeptide therapy. Elevated meropenem MICs suggest that carbapenems may also be unreliable. In the absence of formal treatment guidelines for these rare pathogens, we recommend susceptibility-guided therapy, reinforcing the importance of early identification with MALDI-TOF-MS and rapid susceptibility testing. Second, prolonged and/or broad-spectrum antimicrobial therapy selects for pathogens with reduced susceptibility, particularly in immunocompromised hosts with disrupted gut mucosa and poor source control. Consequently, *Pediococcus* spp. and *Leuconostoc* spp. infections may arise more frequently in advanced malignancy and warrant specific consideration in this setting.

## Ethics declaration

Written informed consent to take part in the study and to publish the article has been obtained from all participants or their legal representatives. The privacy rights of participants have been observed.

This study was performed in compliance with relevant laws, regulatory frameworks and guidelines where the research took place. This study was conducted in accordance with National Statement of Ethical Conduct in Human Research. This study was approved by the St John of God Health Care Human Research Ethics Committee. (Approval No. 2401).

This research follows the CARE guidelines and the CARE checklist.

## CRediT authorship contribution statement

**Sudha Pottumarthy-Boddu:** Writing – review & editing, Validation, Resources, Investigation, Formal analysis. **Aung Du:** Writing – review & editing, Writing – original draft, Visualization, Validation, Resources, Project administration, Investigation, Data curation, Conceptualization. **Dipna Martin-Robins:** Writing – review & editing. **Andrew Dean:** Writing – review & editing, Validation, Supervision. **Eliza Briggs:** Writing – review & editing, Validation. **Arindam Chakravorty:** Writing – review & editing, Supervision, Project administration, Investigation, Formal analysis, Conceptualization. **Blathine Tierney:** Writing – review & editing, Validation. **Jacqueline Mazzer:** Writing – review & editing. **Andrew Hart:** Writing – review & editing, Validation. **Shannyn George:** Writing – review & editing, Validation.

## Informed consent and patient details

Informed consent for publication of this case report was obtained from the patient.

## Declaration of generative AI and AI-assisted technologies in the writing process

The authors declare that no generative AI or AI-assisted technologies were used in the preparation of this article.

## Funding

The authors received no external funding.

## Conflict of Interest

The authors declare no competing interests.

## Data Availability

No datasets were generated or analysed in this study.
