# Supplementary material for: Breakthrough Pediococcus acidilactici and Leuconostoc lactis bacteraemia with elevated meropenem minimum inhibitory concentrations during broad-spectrum antimicrobial therapy in a patient with advanced pancreatic cancer: A case report and review of the literature
Source: IDCases. 2026 Jul 18;45:e02692. doi: 10.1016/j.idcr.2026.e02692 (PMC13393348; doi:10.1016/j.idcr.2026.e02692)
Supplement: Supplementary file 1 — Supplementary material [file mmc1.docx]

**Appendix A. Case reports of *Pediococcus acidilactici* bacteraemia.**

| Reference | Age /ASAB | Reason for hospital admission | Risk factors for bacteraemia^[[1]](#footnote-1)^ | Antimicrobial(s) used in the previous 90 days prior to confirmatory blood cultures | Targeted treatment | Carbapenem MIC (mg/L) | Outcome |
| --- | --- | --- | --- | --- | --- | --- | --- |
| Tachikawa et al. [1] | 16 years/M | Haemorrhagic colitis | Leukaemia, immunosuppressant drugs, immunotherapy and haemorrhagic colitis | Sulfamethoxazole-trimethoprim | Resolved without antimicrobials | Not reported | Recovered |
| Suh et al. [2] | 67 years/F | Bacteraemia | Metastatic adenocarcinoma of the gallbladder, cholecystectomy, chemotherapy, radiotherapy, biliary stent and partial small bowel obstruction | Piperacillin-tazobactam, vancomycin, metronidazole, ciprofloxacin and caspofungin | Daptomycin | Not reported | Recovered |
| Iwen et al. [3] | 32 years/M | Infective endocarditis | Explantation of rejected donor small bowel, multiple abdominal surgeries, short gut syndrome, central venous catheter and diabetes mellitus | Piperacillin-tazobactam, vancomycin and micafungin | Daptomycin | Meropenem (2 mg/L) | Recovered |
| Golledge et al. [4] | 53 years/M | Acute leukaemia | None | Vancomycin, ceftazidime, metronidazole and aciclovir | Benzylpenicillin | Not reported | Recovered |
| Sarma & Mohanty [5] | 26 years/F | Pneumonitis and bacteraemia | None | Ceftriaxone | Benzylpenicillin | Not reported | Recovered |
| Barton et al. [6] | 34 weeks/F | Lethargy, irritability and scleral icterus | Gastroschisis repair, bowel resection, central venous catheter and pseudomembranous colitis | Ampicillin, gentamicin, vancomycin, cefotaxime and clindamycin | Ampicillin and gentamicin | Not reported | Recovered |
| Heinz et al. [7] | 29 years/M | Appendicitis | Peritonitis, small intestinal perforation and bleeding, multiple laparotomies, hemicolectomy and partial excision of jejunum and ileum | Piperacillin-tazobactam, ceftazidime, imipenem, tobramycin, ciprofloxacin, vancomycin and fluconazole | Imipenem and clindamycin | Not reported | Recovered |
| Mastro et al. [8] | 62 years/M | Extensive full thickness burns and pelvic fracture | None | Naficillin, gentamicin, vancomycin and tobramycin | Piperacillin-tazobactam | Not reported | Recovered |
|  | 64 years/F | Acute myocardial infarction | Central venous catheter | Ampicillin | Unknown | Not reported | Died |
|  | 65 years/M | Fever, chills and nausea | Pancreatic cancer and head of pancreas resection | Cephalexin and imipenem-cilastatin | Mezlocillin | Not reported | Recovered |
|  | 69 years/M | Cardiopulmonary arrest | Diabetes mellitus | Cefazolin | Oxacillin and gentamicin^[[2]](#footnote-2)^ | Not reported | Died |
|  | 75 years/F | Exacerbation of bronchitis | Diabetes mellitus, cervical cancer and gastrotomy | Cefazolin and gentamicin | Cefazolin and ticarcillin^[[3]](#footnote-3)^ | Not reported | Resolved |
|  | 89 years/M | Transurethral resection of the prostate | None | Cefazolin and penicillin | Sulfamethoxazole-trimethoprim | Not reported | Resolved |
|  | 85 years/M | Aspiration pneumonia | Prostate cancer, bladder cancer and ileal conduit | Cephalexin, ampicillin and sulfamethoxazole-trimethoprim | Ampicillin and gentamicin | Not reported | Resolved |
|  | 81 years/F | Fever, hypotension and decreased mental state | Leukaemia and diabetes mellitus | Mezlocillin, cefazolin, sulfamethoxazole-trimethoprim, gentamicin and vancomycin | Mezlocillin, amikacin, cefazolin, ciprofloxacin^[[4]](#footnote-4)^ | Not reported | Died |
|  | 16 days/F | Vomiting and weight loss | Jejunoileal atresia and jejunoileostomy | Ampicillin and amikacin | Ampicillin and gentamicin | Not reported | Resolved |
| Sire et al. [9] | 51 years/M | Intra-abdominal mass | Laparotomy, partial gastrectomy with jejunocolostomy, colostomy, cholecystectomy and central venous catheter | Ceftazidime, gentamicin, metronidazole, amikacin and vancomycin | Imipenem, fosfomycin and vancomycin^[[5]](#footnote-5)^ | Not reported | Died |

Abbreviations: ASAB, assigned sex at birth; F, female; M, male; MIC, minimum inhibitory concentration; mg/L, milligrams per litre

**Appendix B. Case reports of *Leuconostoc lactis* bacteraemia.**

| Reference | Age/ASAB | Reason for hospital admission | Risk factors for bacteraemia^[[6]](#footnote-6)^ | Antimicrobial(s) used in the previous 90 days prior to confirmatory blood cultures | Targeted treatment | Carbapenem MIC (mg/L) | Outcome |
| --- | --- | --- | --- | --- | --- | --- | --- |
| Swain et al. [10] | 62 years/M | Ten days history of fever | Type 2 diabetes, myelofibrosis, pancytopenia and immunosuppressant drugs | Vancomycin | None | Not reported | Died |
| Yang et al. [11] | 83 years/F | Cholelithasis, obstructive cholangitis, pneumonia and shock | Previous cholecystotomy, central venous catheter and intra-abdominal infection | Meropenem, vancomycin, cefoperazone/sulbactam, minocycline and linezolid | None | Meropenem (≥ 4mg/L) and ertapenem (≥ 8mg/L) | Died |
| Patel et al. [12] | 52 years/F | Chemotherapy | Chemotherapy, granulocytic sarcoma and peripherally inserted central catheter | Meropenem, teicoplanin, linezolid, caspofungin and ciprofloxacin | Tigecycline | Not reported | Recovered |
| Matsuda et al. [13] | 44 years/F | Fever and malaise | Leukaemia and chemotherapy | Cefepime, meropenem, doripenem and teicoplanin | Daptomycin | Doripenem (6 mg/L) | Recovered |
| Omori et al. [14] | 62 years/M | Meningitis | Type 2 diabetes, alcoholic liver cirrhosis, hepatocellular carcinoma and transcatheter arterial chemoembolization of hepatic lesion | Meropenem, vancomycin and ampicillin | Ampicillin | Not reported | Recovered |
| Hosoya et al. [15] | 71 years/M | Boerhaave syndrome | Oesophageal perforation and surgical repair and mediastinitis | None | Piperacillin-tazobactam, followed by ampicillin-sulbactam | Not reported | Unknown |
| Shin et al. [16] | 59 years/F | Tuberculous arthritis | Immunosuppressant drugs, rheumatoid arthritis and tuberculosis | Ciprofloxacin and metronidazole | Piperacillin-tazobactam, followed by ampicillin | Not reported | Died |
| Gagliardo et al. [17] | 3 years/F | Twitching of extremities, eye deviation and oxygen desaturation | Gastrotomy | None | Ampicillin | Not reported | Unknown |
| Azghar et al. [18] | 4 years/F | Four days of fever | Pancytopenia secondary to bone marrow aplasia | None | Ceftriaxone, amoxycillin and gentamicin | Not reported | Recovered |
| Carapetis et al. [19] | 4 years/M | Fever and vomiting | Small bowel resection for volvulus and central venous catheter | Sulfamethoxazole-trimethoprim | Amoxycillin | Not reported | Recovered |
| Kocak et al. [20] | 7 months/U | Arterial switch operation | Central venous catheter | Vancomycin | Cefotaxime | Not reported | Died |
| Vagiakou-Voudris et. al [21] | 52 years/M | Septic shock | Type 2 diabetes | Ciprofloxacin, netilmicin and metronidazole | Imipenem, neilmicin and amoxycillin-clavulanate | Not reported | Recovered |
| Botan et al. [22] | 1 day/U | Lung infection | None | Ampicillin and gentamicin | Ampicillin and gentamicin | Not reported | Recovered |
| Deng et al. [23] | 45 years/M | Abdominal discomfort, jaundice and fever following liver transplant | Previous liver transplant, immunosuppressant drugs and central venous catheter | Ceftazidime and vancomycin | Linezolid^[[7]](#footnote-7)^ | Not reported | Recovered |
| Švec et al. [24] | 55 years/M | Polytrauma | Unknown | Unknown | Piperacillin-tazobactam and ciprofloxacin | Not reported | Unknown |
|  | 6 months/F | Short bowel syndrome | Ileus and central venous catheter | Unknown | Amoxycillin-clavulanate | Not reported | Recovered |
| Bush et al. [25] | 33 years/M | Induction chemotherapy for leukaemia | Leukaemia, infusion port, chemotherapy and neutropenia | Cefepime, vancomycin, meropenem, amphotericin B and vancomycin | Meropenem and daptomycin | Not reported | Unknown |
| Caso et al. [26] | 72 years/F | Acute pancreatitis | Central venous catheter | Glycopeptide^[[8]](#footnote-8)^ | Tigecycline | Not reported | Recovered |

Abbreviations: ASAB, assigned sex at birth; F, female; M, male; U, unknown; MIC, minimum inhibitory concentration; mg/mL, milligrams per litre

**References**

[1] Tachikawa J, Aizawa Y, Izumita R, Shin C, Imai C, Saitoh A. Resolution of Pediococcus acidilactici bacteremia without antibiotic therapy in a 16-year-old adolescent with leukemia receiving maintenance chemotherapy. IDCases 2022;27:e01384. https://doi.org/10.1016/J.IDCR.2022.E01384.

[2] Suh B. Resolution of persistent Pediococcus bacteremia with daptomycin treatment: case report and review of the literature. Diagn Microbiol Infect Dis 2010;66:111–5. https://doi.org/10.1016/J.DIAGMICROBIO.2008.10.003.

[3] Iwen PC, Mindru C, Kalil AC, Florescu DF. Pediococcus acidilactici Endocarditis Successfully Treated with Daptomycin. J Clin Microbiol 2012;50:1106. https://doi.org/10.1128/JCM.05648-11.

[4] Golledge CL, Stingemore N, Aravena M, Joske D. Septicemia caused by vancomycin-resistant Pediococcus acidilactici. J Clin Microbiol 1990;28:1678–9. https://doi.org/10.1128/JCM.28.7.1678-1679.1990.

[5] Sarma PS, Mohanty S. Pediococcus acidilactici Pneumonitis and Bacteremia in a Pregnant Woman. J Clin Microbiol 1998;36:2392. https://doi.org/10.1128/JCM.36.8.2392-2393.1998.

[6] Barton LL, Rider ED, Coen RW. Bacteremic Infection With Pediococcus: Vancomycin-Resistant Opportunist. Pediatrics 2001;107:775–6. https://doi.org/10.1542/PEDS.107.4.775.

[7] Heinz M, Von Wintzingerode F, Moter A, Halle E, Lohbrunner H, Kaisers U, et al. A case of septicemia with Pediococcus acidilactici after long-term antibiotic treatment. Eur J Clin Microbiol Infect Dis 2000;19:946–8. https://doi.org/10.1007/S100960000409.

[8] Mastro TD, Spika JS, Lozano P, Appel J, Facklam RR. Vancomycin-resistant Pediococcus acidilactici: nine cases of bacteremia. J Infect Dis 1990;161:956–60. https://doi.org/10.1093/INFDIS/161.5.956.

[9] Sire JM, Donnio PY, Mesnard R, Pouëdras P, Avril JL. Septicemia and hepatic abscess caused by Pediococcus acidilactici. Eur J Clin Microbiol Infect Dis 1992;11:623–5. https://doi.org/10.1007/BF01961670.

[10] Swain B, Sahu K, Rout S. Leuconostoc lactis: An unusual cause for bacteremia. CHRISMED Journal of Health and Research 2015;2:367. https://doi.org/10.4103/2348-3334.165747.

[11] Yang C, Wang D, Zhou Q, Xu J. Bacteremia Due to Vancomycin-Resistant Leuconostoc lactis in a Patient With Pneumonia and Abdominal Infection. Am J Med Sci 2015;349:282. https://doi.org/10.1097/MAJ.0000000000000380.

[12] Patel T, Molloy A, Smith R, Balakrishnan I. Successful Treatment of Leuconostoc Bacteremia in a Neutropenic Patient with Tigecycline. Infectious Disease Reports 2012, Vol 4, Page E31 2012;4:e31. https://doi.org/10.4081/IDR.2012.E31.

[13] Matsuda K, Koya J, Toyama K, Ikeda M, Arai S, Nakamura F, et al. A therapeutic benefit of daptomycin against glycopeptide-resistant gram-positive cocci bloodstream infections under neutropenia. J Infect Chemother 2017;23:788–90. https://doi.org/10.1016/J.JIAC.2017.06.010.

[14] Omori R, Fujiwara S, Ishiyama H, Kuroda H, Kohara N. Leuconostoc lactis- A Rare Cause of Bacterial Meningitis in an Immunocompromised Host. Internal Medicine 2020;59:2935–6. https://doi.org/10.2169/INTERNALMEDICINE.5076-20.

[15] Hosoya S, Kutsuna S, Shiojiri D, Tamura S, Isaka E, Wakimoto Y, et al. Leuconostoc lactis and Staphylococcus nepalensis Bacteremia, Japan - Volume 26, Number 9—September 2020 - Emerging Infectious Diseases journal - CDC. Emerg Infect Dis 2020;26:2283–5. https://doi.org/10.3201/EID2609.191123.

[16] Shin J, Her M, Moon C, Kim D, Lee S, Jung S. Leuconostoc bacteremia in a patient with amyloidosis secondary to rheumatoid arthritis and tuberculosis arthritis. Mod Rheumatol 2011;21:691–5. https://doi.org/10.3109/S10165-011-0465-0.

[17] Gagliardo C, Johnson E, Di Pentima MC. LEUCONOSTOC LACTIS SEPSIS IN A CHILD WITH CHROMOSOMAL 18 ABNORMALITY RECEIVING ENTERAL NUTRITION. J Paediatr Child Health 2021;57:170. https://doi.org/10.1111/JPC.15236;PAGEGROUP:STRING:PUBLICATION.

[18] Azghar A, Azizi M, Lahmer M, Benaissa E, Lahlou Y Ben, Benajiba N, et al. A very rare case of bacteraemia in a 4-year-old girl with osteopetrosis with probable Leuconostoc lactis infection. Access Microbiol 2023;5:000439. https://doi.org/10.1099/ACMI.0.000439.

[19] Carapetis J, Bishop S, Davis J, Bell B, Hogg G. Leuconostoc sepsis in association with continuous enteral feeding: two case reports and a review. Pediatr Infect Dis J 1994;13:816–23. https://doi.org/10.1097/00006454-199409000-00014.

[20] Koçak F, Yurtseven N, Aydemir N, Yüksek A, Yavuz SŞ. A case of osteomyelitis due to Leuconostoc lactis. Scand J Infect Dis 2007;39:278–80. https://doi.org/10.1080/00365540600904811.

[21] Vagiakou-Voudris E, Mylona-Petropoulou D, Kalogeropoulou E, Chantzis A, Chini S, Tsiodra P, et al. Multiple liver abscesses associated with bacteremia due to Leuconostoc lactis. Scand J Infect Dis 2002;34:766–7. https://doi.org/10.1080/00365540260348572.

[22] Botan E, Aydin B, Gultekin C, Botan E, Aydin B, Gultekin C, et al. Leuconostoc Lactis as an Early-onset Neonatal Sepsis Agent: A Case Report with the Current Literature Review. The Medical Bulletin of Haseki 2023;61:69–71. https://doi.org/10.4274/HASEKI.GALENOS.2023.8611.

[23] Deng Y, Zhang Z, Xie Y, Xiao Y, Kang M, Fan H. A mixed infection of Leuconostoc lactis and vancomycin-resistant Enterococcus in a liver transplant recipient. J Med Microbiol 2012;61:1621–4. https://doi.org/10.1099/JMM.0.045161-0.

[24] Švec P, Ševčíková A, Sedláček I, Bednářová J, Snauwaert C, Lefebvre K, et al. Identification of lactic acid bacteria isolated from human blood cultures. FEMS Immunol Med Microbiol 2007;49:192–6. https://doi.org/10.1111/J.1574-695X.2006.00199.X.

[25] Bush LM, Williams J. Leuconostoc lactis Bacteremia and Neutropenic Fever: An Infrequently Encountered Vancomycin-Resistant Gram-Positive Cocci: A Case Report and Review. Infectious Diseases in Clinical Practice 2023;31. https://doi.org/10.1097/IPC.0000000000001234.

[26] Caso JM, Recio R, Ruiz-Ruigómez M, Orellana MÁ, Fernández-Ruiz M. Bacteremia due to Leuconostoc species: A 13-year single-center case series. Enferm Infecc Microbiol Clin 2024;42:149–51. https://doi.org/10.1016/J.EIMC.2023.01.003.

1. Includes any abdominal surgeries, bowel pathologies that increase gastrointestinal mucosa permeability, immunosuppressive states and central venous catheters [↑](#footnote-ref-1)
2. Treated for concurrent bacteraemia with *Staphylococcus epidermidis* [↑](#footnote-ref-2)
3. Treated for concurrent gastrotomy wound with *Pseudomonas aeruginosa* [↑](#footnote-ref-3)
4. Treated for concurrent bacteraemia with *Staphylococcus aureus* [↑](#footnote-ref-4)
5. Treated for concurrent bacteraemia with *Enterococcus faecalis* and *Pseudomonas aeruginosa* [↑](#footnote-ref-5)
6. Includes any abdominal surgeries, bowel pathologies that increase gastrointestinal mucosa permeability, immunosuppressive states and central venous catheters [↑](#footnote-ref-6)
7. Treated for concurrent intra-abdominal infection with *Enterococcus faecium* [↑](#footnote-ref-7)
8. Antimicrobial not specified [↑](#footnote-ref-8)
